# Comparative evaluation of epidural bupivacaine alone and bupivacaine combined with magnesium sulfate in providing postoperative analgesia: a meta-analysis of randomized controlled trials

**DOI:** 10.1186/s12871-020-0947-8

**Published:** 2020-02-05

**Authors:** Li-Qin Li, Mei-Dan Fang, Cong Wang, Hong-Liu Lu, Li-Xue Wang, Hong-Yu Xu, Hou-Zhong Zhang

**Affiliations:** 1 Department of Anesthesiology, Guizhou Provincial People’s Hospital, Guiyang, Guizhou People’s Republic of China; 2 Department of Anesthesiology, Jilin University Second Hospital, No. 218 Ziqiang street, Changchun, Jilin, 130021 People’s Republic of China; 3 Department of Anesthesiology, First Affiliated Hospital to Changchun University of Chinese Medicine, Changchun, Jilin, People’s Republic of China

**Keywords:** Magnesium sulfate, Postoperative analgesia, Epidural anesthesia, Bupivacaine

## Abstract

**Background:**

The comparative efficacy of epidural bupivacaine alone and bupivacaine combined with magnesium sulfate in providing postoperative analgesia remains controversial.

**Methods:**

We searched Mediline (OvidSP), EMBASE (OvidSP) and Cochrane Central Register of Controlled Trials (CENTRAL) to identify trials that compared epidural bupivacaine and magnesium sulfate combination (intervention) with bupivacaine alone (control). Grading of Recommendations, Assessment, Development and Evaluations (GRADE) framework was used to assess the quality of evidence.

**Results:**

Eleven studies fulfilled our inclusion criteria after screening. We found that epidural bupivacaine combined with magnesium sulfate could prolong the time for first rescue analgesics (SMD 4.96; 95% CI [2.75, 7.17], *P* < 0.00001, I^2^ = 98%), reduce the number of patients who need rescue analgesics (RR 0.38; 95% CI [0.20, 0.74], *P* = 0.004, I^2^ = 75%) and requirement for rescue analgesics (SMD -2.65; 95% CI [− 4.23, − 1.06], *P* = 0.001, I^2^ = 96%).

**Conclusions:**

Magnesium suifate as an adjuvant of epidural bupivacaine improved postoperative analgesia. However, we rated the quality of evidence to be very low because of high heterogeneity, imprecise of results and small sample sizes. Furthermore, further large high-quality trials are still needed to confirm the effects of magnesium sulfate on postoperative analgesia.

## Background

Alleviation of postoperative pain is an important objective for the anesthesiologists. Inadequaten postoperative pain control is associated with deep vein thrombosis and pulmonary embolism, quality-of-life impairment, delayed recovery time and higher health-care costs [1, 2]. Epidural anaesthesia is an effective technique, with the advantage of safety, efficiency and prolonged postoperative pain relief [3]. A number of adjuvants have been used with bupivacaine via epidural route over the years with the purpose of prolonging the duration of postoperative analgesia and minimising the side effects. Nevertheless researchers continue to find the optimum adjuvants, as the currently researched adjuvants (e.g. opioids, tramadol, dexmedetomidine) still have some adverse effects such as nausea and vomiting, pruritus, bradycardia, and hypotension [4–6].

Magnesium is the fourth most plentiful cation in the body and possess certain analgesic property in both animal and human models of pain [7, 8]. Magnesium ion (Mg2+) is a non-competitive N-methyl-D-aspartate (NMDA) receptor antagonist that blocks inward current flow through ion channels linked to NMDA receptors in a voltage-dependent fashion, has the potential to prevent central sensitization induced by peripheral nociceptive stimulation. For these reasons, it seems plausible that magnesium as an adjuvant of epidural bupivacaine can prolong postoperative analgesia and reduce side effects. Therefore we conducted this meta-analysis to test our hypothesis.

## Materials and methods

For this meta-analysis, we followed the recommendation with Preferred Reporting Items for Systematic Reviews and Meta-Analyses (PRISMA) guidelines [9], and the quality of the evidence was assessed using the GRADE approach and recommendations from the Cochrane Collaboration [10].

### Eligibility and exclusion criteria

The purpose of this meta-analysis was to evaluate the safety and efficacy of magnesium sulfate as an adjuvant of epidural bupivacaine. Studies will be included if they meet the following criteria: (1) randomized controlled trials (RCTs); (2) adults (≥18 years old); (3) comparing the analgesic efficacy of epidural bupivacaine and magnesium sulfate combination (intervention), with bupivacaine alone (control) for anesthesia or postoperative pain management; (4) study provided data at least on one of the outcomes (time to the first rescue analgesia, number of patients required rescue analgesia, requirement for rescue analgesia, duration of motor block, and side effects); (5) full text published in English. We excluded studies in which another drug (eg.fentanyl, morphine) was added in the intervention or control group, and magnesium sulfate was administered by another route (e.g. intrathecal, intravenous or intramuscular).

### Endpoints

Primary outcomes: (1) the time to the first request for rescue analgesics; (2) the number of patients required postoperative rescue analgesics; (3) requirement for rescue analgesics. Secondary outcomes: (1) duration of motor block; (2) adverse events related to postoperative analgesia protocols (the incidence of hypotension, bradycardia, nausea and vomiting, pruritus, shivering).

### Search strategy and study selection

We searched Mediline (OvidSP), EMBASE (OvidSP) and Cochrane Central Register of Controlled Trials (CENTRAL) on october 24, 2019 in order to identify trials that compared epidural bupivacaine and magnesium sulfate combination (intervention) with bupivacaine alone (control). The exact search strategies are shown in Appendix 1. After importing the search results into EndNote X9, duplicated studies were excluded. Two investigators (Li and Wang) independently determined eligibility on the basis of the title, abstract and full text according to the inclusion and exclusion criteria, with disagreements resolved by discussion and consensus with a third agent (Fang).

### Data extraction and quality assessment

Two review authors (Li and Wang), independently extracted data using a predesigned form and verified for consensus before entry into Review Manager 5.0. Li and Wang independently assessed the risk of bias for each included study using the criteria outlined in the Cochrane Handbook for Systematic Reviews of Interventions [11]. We rated the overall risk of bias of a study as low if ≦1 domain were ‘high risk’ or ‘unclear’, high if two or more of the domains were identified as ‘high risk’ or ‘unclear’. Resolving any disagreements by discussion and consensus with a third reviewer (Fang).

### Statistical analysis

We used the software Review Manager 5.0 for statistical analysis. Only primary and secondary outcomes defined previously were included in our analysis. For continuous data (e.g. the time to the first request for rescue analgesics, requirement for rescue analgesics and duration of motor block), considering the different modes of postoperative pain management and species of rescue analgesics, we calculated standardized mean differences (SMDs) with corresponding 95% confidence intervals (95% CI). We rated the effect size of SMDs as small effect (0.2–0.5), medium effect (0.5–0.8) and large effect (≥0.8). For those studies that did not report a mean and standard deviation (SD), we did not hand and transformate the date, because we did not know if it was normally distributed. We calculated risk ratios (RR) with corresponding 95% CI for dichotomous outcomes (e.g. the number of patients required postoperative rescue analgesics, adverse events related to postoperative analgesia protocols).

### Assessment of heterogeneity and subgroup analysis

We assessed statistical heterogeneity using the I^2^ statistic, as described in the Cochrane Handbook for Systematic Reviews of Interventions [11], and an I^2^ value > 50% is considered to indicate substantial heterogeneity. To explore the sources of clinical heterogeneity, we planned to perform subgroup analyses of bolus injection versus bolus followed a continuous injection according to the administration of magnesium sulfate. We used a randomized-effect model if there was significant heterogeneity among studies (I^2^ > 50%), otherwise the fixed effects model was used. A *P*-value < 0.05 and the 95% CI did not cross the equivalent line were considered statistically significant differences from control.

### Sensitivity analysis and assessment of publication bias

We decided to perform sensitivity analyses for the primary outcomes by removing studies with high risk of of bias or using two different models (the randomized effect model and the fixed effect model). If sufficient studies (10 or more) were included for the primary or second outcomes, we had intended to use a funnel plot to explore the possibility of publication bias.

## Results

### Study selection and characteristics of included studies

**Fig. 1:**
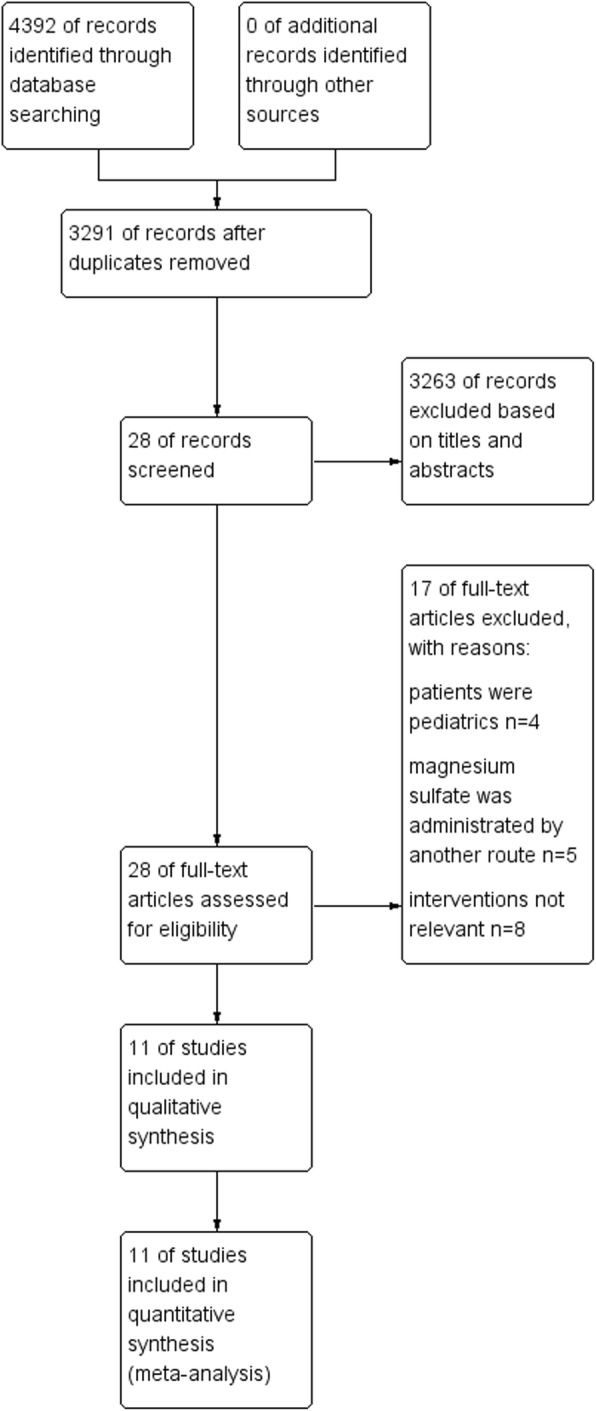
The details of retrieval

The literature search identified 4392 studies totally and 112 references from CENTRAL, 2481 references from MEDLINE (OvidSP), and 1799 studies from Embase (Ovidsp). Eleven studies fulfilled our inclusion criteria after screening [12–22]. The details of retrieval was shown in Fig. 1. The various characteristics of the included studies were shown in Table 1. Seven trials applied magnesium sulfate as a bolus followed by a continuous infusion [12–18], while the remaining four trials applied a bolus injection [19–22]. In eight studies enrolled participants undergoing Lower abdominal or lower limb surgeries [12, 13, 15, 17–20, 22], with participants administered epidural anesthesia. Participants in Mohammad 2015 and Radwan 2017 underwent unilateral thoracic surgery and spine surgery respectively with general plus epidural anesthesia. Two studies enrolled participants undergoing cesarean section, with participants in Sun 2012 administered combines spinal epidural anesthesia, and participants in Elsharkawy 2018 administered epidural anesthesia. Total enrollment ranged from 40 to 100 participants, with the number of participants in each study epidural bupivacaine and magnesium sulfate combination ranging from 20 to 50, control 20 to 50.

**Table 1:** Characteristics of included trials

Study	Patients (MgSO4/control)	Surgery	Anesth-esia strateg-y	Intervention	Control	Postoperative analgesia
Aly 2011	30/30	Orthoped-ic surgeries	EA	bupivacaine 0.5% + 50 mg magnesium sulphate (MgSO4) in 10 ml as an initial bolus dose followed by infusion of 10 mg/ h (2 ml/h) during the surgery.	N/S in same volume	When surgery was completed, all patients received PCEA using a PCEA device containing fentanyl 2 μg/ml and bupivacaine 0.03% (0.3 mg/ml). The PCEA was programmed to deliver 2.5 ml/h infusion with a bolus dose of 1.5 ml on demand. The lockout interval between boluses was 6 min. If patients had inadequate analgesia, supplementary rescue analgesia with intramuscular pethidine 50 mg was available.
Daabiss 2013	40/40	Total knee replacem-ent surgery	EA	Bupivacaine 0.5% + 50 mg magnesium sulphate (MgSO4) in 10 ml as an initial bolus dose followed by infusion of 10 mg/h (diluted in 10 ml saline) during the surgery.	N/S in same volume	When surgery was complete, all patients received PCEA using a PCEA device containing fentanyl 2 μg/ml and bupivacaine 0.08%. The PCEA was programmed to administer a demand bolus dose of fentanyl 5 ml with no background infusion and lockout interval 20 min. If patients had inadequate analgesia, supplementary rescue analgesia with intramuscular pethidine 50 mg was available.
Elshark-awy 2018	30/30	Cesarean section	EA	Epidural 20 ml levobupivacaine hydrochloride 0.5% followed by 5 ml of 10% preservative-free MgSO4(500 mg) prepared in two separate syringes	N/S in same volume	If the recorded VAS was ≥3, the patient was given diclofenac potassium 75 mg oral tablets as the first rescue analgesia every 12 h. If the VAS was still > 3 within 30 min, patients were given incremental dose of 0.5 μg/kg fentanyl.
Ghatak 2010	30/30	Lower abdomina-l and lower limb surgeries	EA	Bupivacaine 0.5% (19 ml) + magnesium sulphate 50 mg (in 1 ml 0.9% saline)	N/S in same volume	In the event of pain, (VAS ≥40), both intraoperatively as well as postoperatively, a bolus of epidural bupivacaine 0.25% (8 ml) was administered by the anaesthesiologist inside the operation theatre and the nursing staff in the recovery room
Gupta 2013	30/30	Total abdomina-l hysterectomies	EA	1 ml of 50 mg/ml magnesium sulphate in 10 ml epidural anaesthetic solution (9 ml 0.125% bupivacaine)	N/S in same volume	If patients had inadequate analgesia (VAS > 3), epidural fentanyl 1mcg/kg in 10 ml normal saline was administered as supplementary rescue analgesic.
Moham-mad 2015	20/20	Unilatera-l thoracic surgery	GA + EA	Toward the end of the surgery, approximately 20 min prior to anticipated extubation. Bupivacaine 0.25% 8 ml + magnesium sulfate 50 mg in 1 ml 0.9% saline.	N/S in same volume	Epidural infusion with 5 ml/h of 0.1% bupivacaine was started 15 min after the bolus dose, and it was continued during the postoperative period via epidural catheter using an infusion pump. Patients complaining of pain in the postoperative period with VAS score ≥ 4, received tramadol 50 mg IV as rescue analgesia and time to the first request for analgesia was noted.
Omar 2018	50/50	Infraumb-ilical abdomina-l and pelvic surgeries	EA	15 ml mixture of 14 ml levobupivacaine 0.5% + 0.5 ml MgSO410% (50 mg) + 0.5 ml of 0.9 NaCl in epidural catheter at induction then continuous epidural infusion of this mixture by 5 ml/h till the end of the surgery	N/S in same volume	If patients had inadequate analgesia (i.e.,: if VAS ≥4 when measured each hour or if patient expressed intolerable pain in between VAS measurements periods) supplementary rescue analgesic was used, pethidine 1 mg/kg i/m/and paracetamol (Perfalgan®) 1 g i.v. drip.
Radwan 2017	22/22	Spine surgeries	GA + EA	14 ml levobupivacaine 0.5% + 50 mg magnesium sulphate in 1 ml saline. Subsequently continuous epidural infusion of levobupivacaine 0.125% + 2 mg/ml magnesium sulphate. Drugs were prepared in 20 cc syringe and the rate of infusion in each group was 5 ml/h.	N/S in same volume	One gram of iv paracetamol was given to the patients when VAS > 3 and every 8 h thereafter. A second rescue analgesic in the form of 50 mg iv meperidine was given to patients if VAS remained > 3 one hour after iv paracetamol.
Shruthi 2016	20/20	Infraumb-ilical surgery	EA	15 ml of bupivacaine 0.5% + 50 mg of magnesium sulphate (MgSO4) made up to 1 ml.	N/S in same volume	Postoperative analgesia was managed with epidural bolus of bupivacaine 0.125% 8 ml boluses and/or Paracetamol 1 g infusion as per discretion of treating consultants.
Shahi 2014	40/40	Lower limb surgery	EA	Bupivacaine 0.5% (14 ml) + magnesium sulfate 50 mg (in 1 ml 0.9% saline)	N/S in same volume	In the event of pain, (VAS ≥40), both intraoperatively as well as postoperatively, a bolus of epidural bupivacaine 0.125% (12 ml) was administered by the anesthesiologist inside the operation theatre and the nursing staff in the recovery room.
Sun 2012	50/50	Cesarean section	CSEA	After delivery of the fetus, received 0.1% bupivacaine 10 mL and Mg 500 mg.	Nothing added	Postoperatively, oral diclofenac 12.5 mg was given for rescue analgesia whenever the VAS pain score was > 3 cm

*CSEA* Combines spinal epidural anesthesia, *EA* Epidural anesthesia, *GA* General anesthesia, *N/S* Normal saline, *VAS* Visual analogue scale

### Risk of Bias of the included trials

**Fig. 2:**
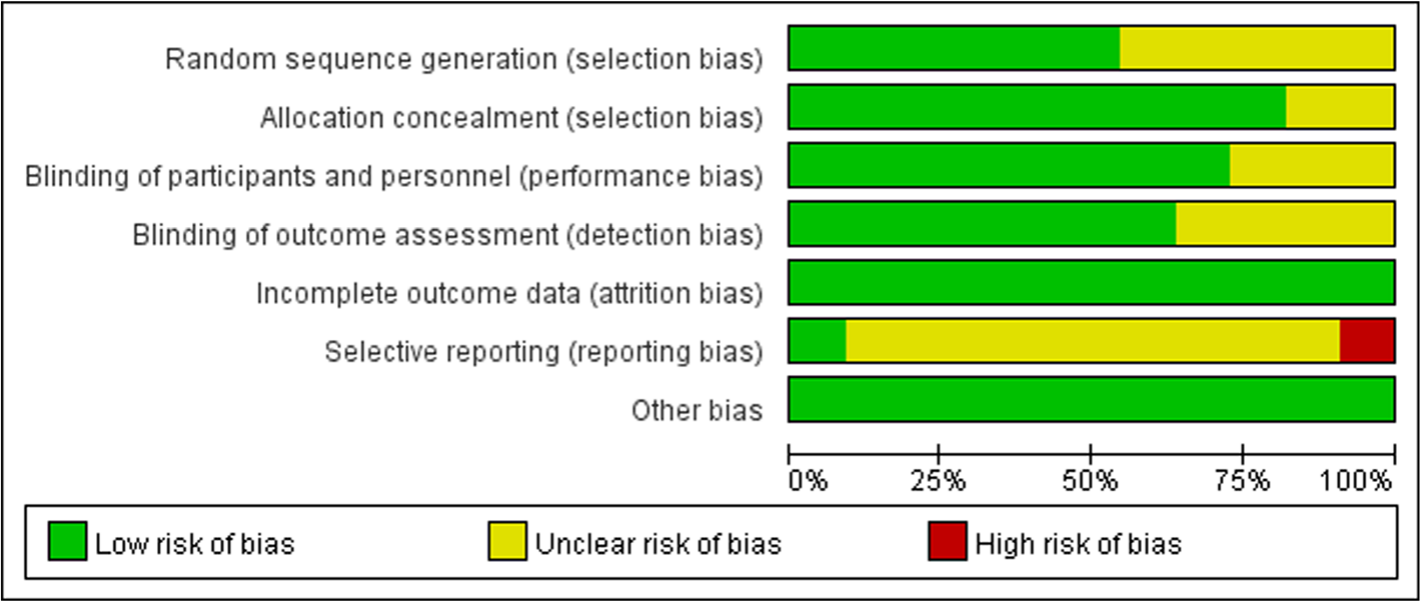
Risk of bias graph. Review authors’ judgements about each risk of bias item presented as percentages across all included studies

**Fig. 3:**
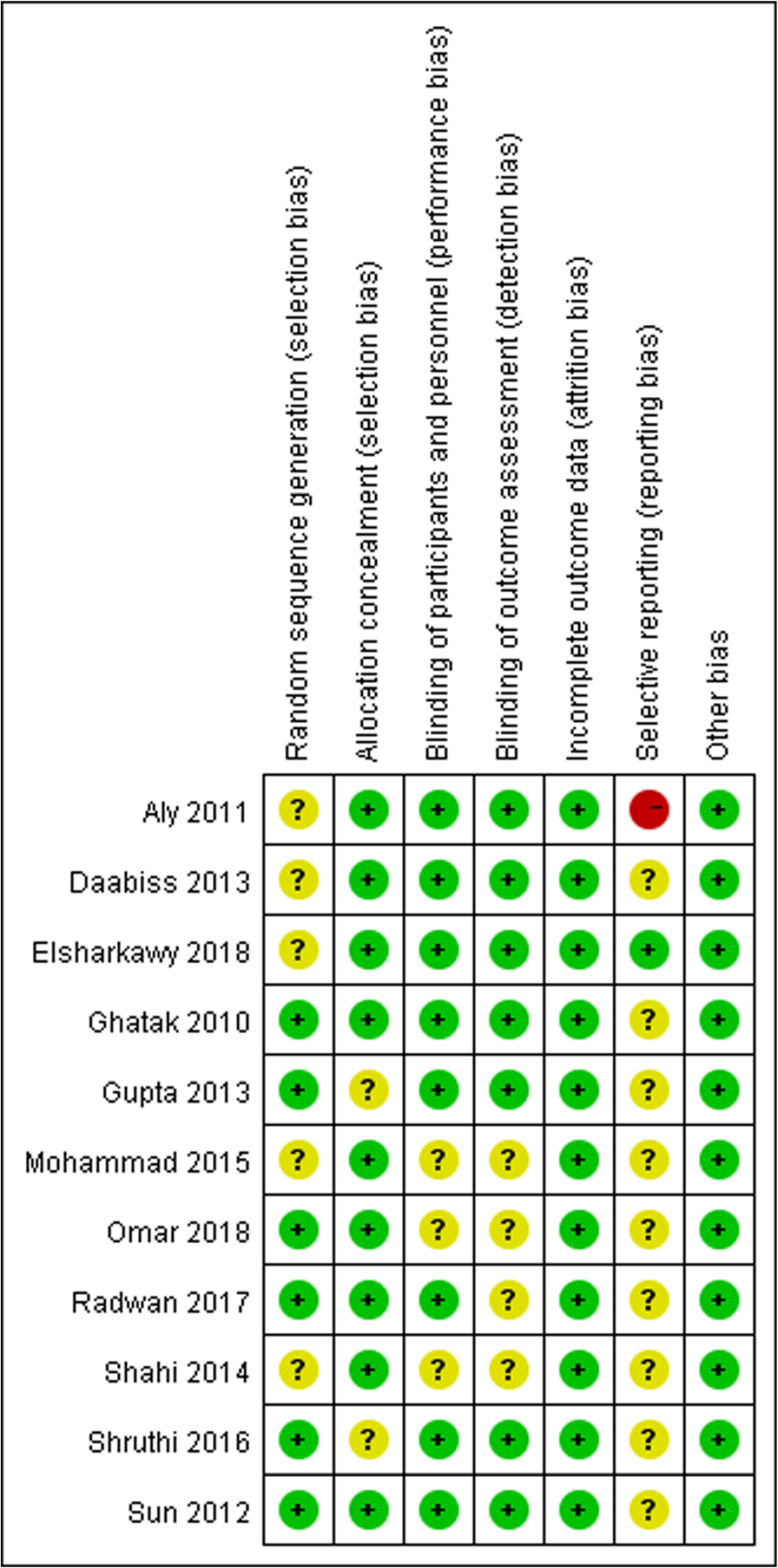
Risk of bias summary: review authors’ judgements about each risk of bias item for each included study

Fig. 2 and Fig. 3 presents the risk of bias of the included studies. We assessed 3 of these trials as low risk of bias [12, 13, 16], while the remaining 8 trials as high risk of bias according to our pre-specified criteria [14, 15, 17–22].

### Primary outcome: the time to the first request for rescue analgesics

**Fig. 4:**
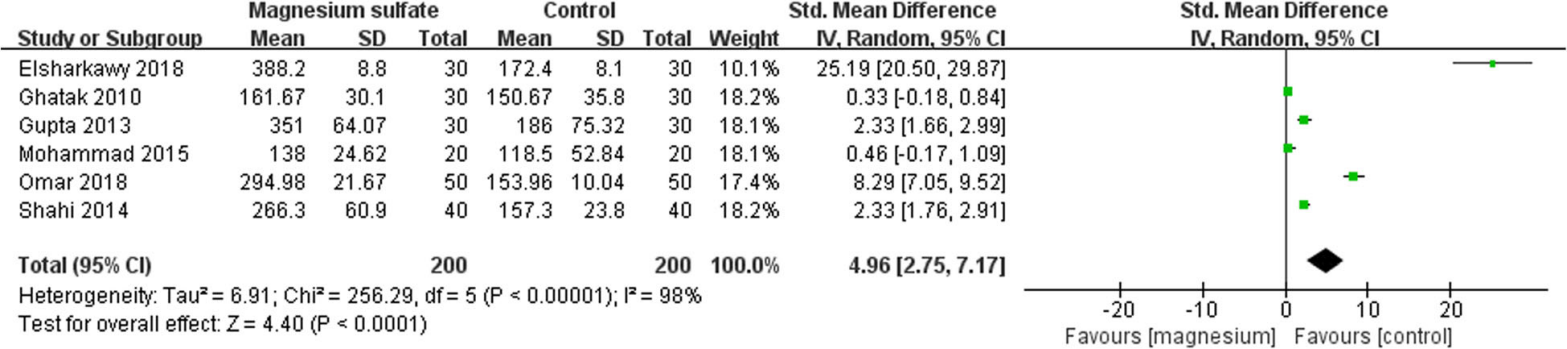
Forest plot of comparison: Magnesium sulfate vs Control, Outcome: the time to the first request for rescue analgesics

Nine studies reported the time to the first request for rescue analgesics, in which the data of six studies were reported as mean ± standard deviation (SD) [12–15, 17, 20], while the remaining two studies reported the data in fig [16, 19, 22].. As a result, only six studies involving 400 patients were included for analysis [12–15, 17, 20]. The meta-analysis showed that the time to the first request for rescue analgesics was prolonged significantly in the magnesium sulfate group compared with the control group (SMD 4.96; 95% CI [2.75, 7.17], *P* < 0.00001, I^2^ = 98%; Fig. 4). We deemed the quality of the evidence to be very low because: (1) four studies had ‘high’ risk of bias; (2) the result was imprecise (wide confidence interval); and (3) there was significant heterogeneity among studies.

Subgroup analysis was conducted according to the administration of magnesium sulfate. Five studies administrated magnesium sulfate by bolus injection [12–15, 17], and only one study by bolus followed a continuous injection [20]. We noted the time to the first request for rescue analgesics was also prolonged significantly in the magnesium sulfate group compared with the control group when the study by bolus followed a continuous injection was excluded (SMD 3.67; 95% CI [1.75, 5.58], *P* = 0.0002, I^2^ = 97%). We also found significant difference between the subgroups (*P* < 0.0001) and the bolus followed a continuous injection subgroup had a greater effect on the time to the first rescue analgesics.

### Primary outcome: the number of patients required postoperative rescue analgesics

**Fig. 5:**
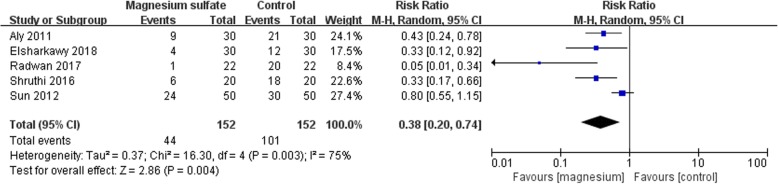
Forest plot of comparison: Magnesium sulfate vs Control, Outcome: the number of patients required rescue analgesics

Five studies including 304 patients reported the number of patients required rescue analgesics [12, 16, 18, 19, 21]. The merged effect analysis showed that it was significantly less in magnesium group compared with control group (RR 0.38; 95% CI [0.20, 0.74], *P* = 0.004, I^2^ = 75%, Fig. 5). We judged, the quality of the evidence to be very low based on the GRADE framework: (1) three studies had a ‘high’ risk of bias; (2) there was significant heterogeneity among studies.

Subgroup analysis was conducted according to the administration of magnesium sulfate. Patients required postoperative rescue analgesics was significantly less in magnesium group when magnesium sulfate was administrated by bolus injection (RR 0.49; 95% CI [0.24, 0.97], *P* = 0.04, I^2^ = 70%). No significant difference was shown between the two groups when magnesium sulfate was administrated by bolus followed a continuous injection (RR 0.17; 95% CI [0.01, 1.97], *P* = 0.16, I^2^ = 84%).

### Primary outcome: requirement for rescue analgesics

**Fig. 6:**
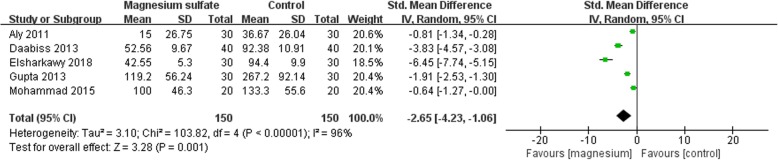
Forest plot of comparison: Magnesium sulfate vs Control, Outcome: the requirement for rescue analgesia

The requirement for rescue analgesia was reported in five studies involving 300 participants [12, 14, 17, 19, 22]. The meta-analysis showed that the magnesium sulfate group had significantly lower consumption of rescue analgesics than the control group (SMD -2.65; 95% CI [− 4.23, − 1.06], *P* = 0.001, I^2^ = 96%; Fig. 6). We rated the quality of the evidence to be very low: (1) four studies had ‘high’ risk of bias; (2) the result was imprecise; and (3) there was significant heterogeneity among studies.

Subgroup analysis was conducted according to the administration of magnesium sulfate. A significant reduction was shown in the magnesium group when magnesium sulfate was administrated by bolus injection (SMD -2.92; 95% CI [− 5.43, − 0.42], *P* = 0.02, I^2^ = 97%). No significant difference was shown between two groups when magnesium sulfate was administrated by bolus followed a continuous injection (SMD -2.31; 95% CI [− 5.26, 0.65], *P* = 0.13, I^2^ = 98%).

### Secondary outcome: duration of motor block

**Fig. 7:**
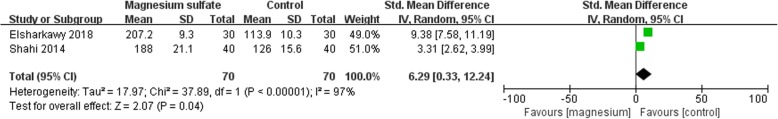
Forest plot of comparison: Magnesium sulfate vs Control, Outcome: the duration of motor block

Two studies reported duration of motor block [12, 15]. The result showed that the duration of motor block in magnesium sulfate group was significantly longer than contol group (SMD 6.29; 95% CI [0.33, 12.24], *P* = 0.04, I^2^ = 97%; Fig. 7). We rated the quality of the evidence to be very low: (1) the result was imprecise; (2) there was significant heterogeneity among studies; and (3) limited availability of evidence.

### Secondary outcome: adverse events related to postoperative analgesia protocols

**Fig. 8:**
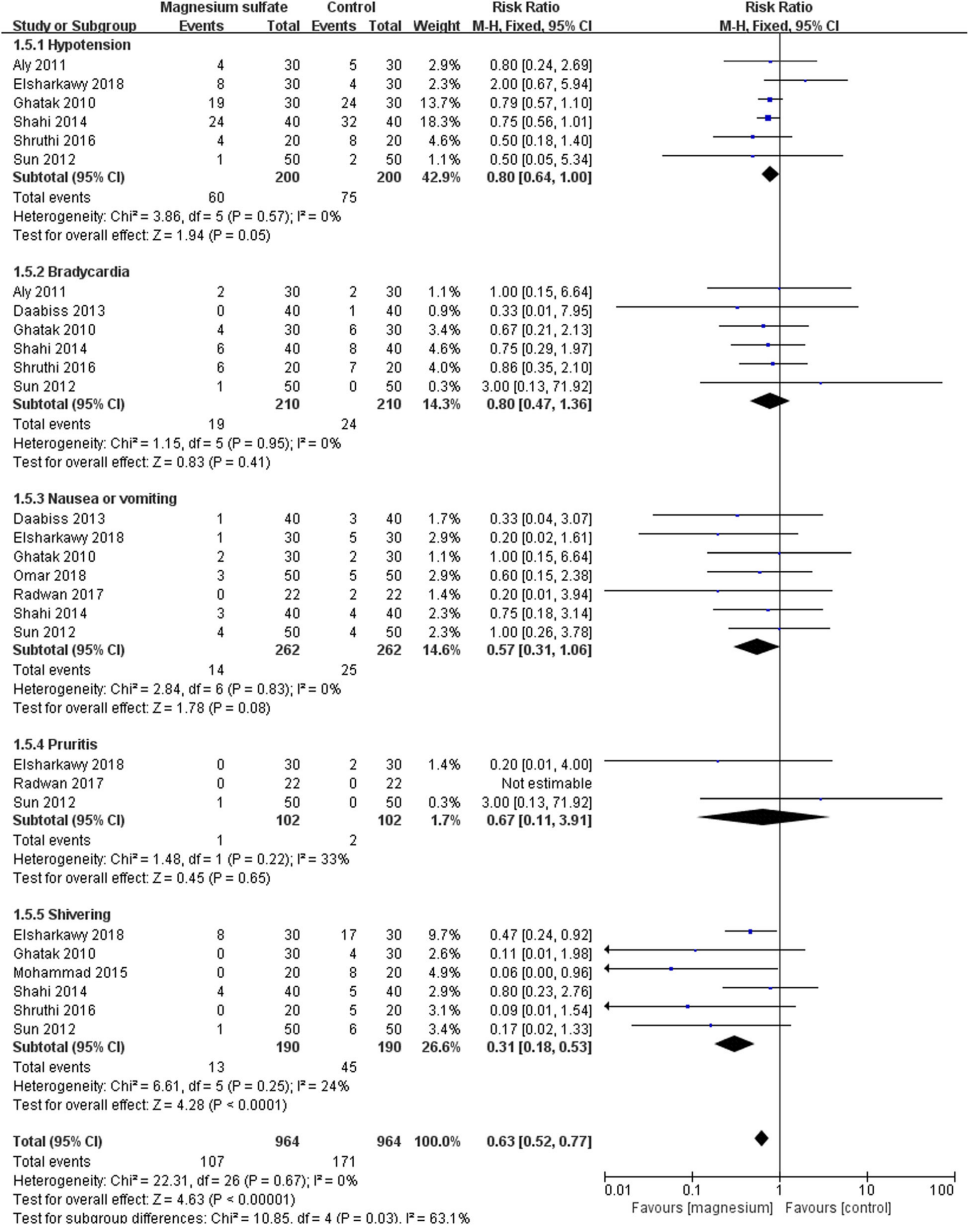
Forest plot of comparison: Magnesium sulfate vs Control, Outcome:the adverse events

A meta-analysis of adverse events was shown in Fig. 8, such as hypotension (RR 0.80; 95% CI [0.64,1.00], *P* = 0.05, I^2^ = 0%), bradycardia (RR 0.80; 95% CI [0.47, 1.36], *P* = 0.41, I^2^ = 0%), nausea and vomiting (RR 0.57; 95% CI [0.31, 1.06], *P* = 0.08, I^2^ = 0%) and pruritus (RR 0.67; 95% CI [0.11, 3.91], *P* = 0.65, I^2^ = 0%) showed no statistically significant differences, except that the incidence of shivering in magnesium sulfate group was significantly lower than control group (RR 0.31; 95% CI [0.18, 0.53], *P* < 0.0001, I^2^ = 24%). We rated the evidence for adverse events to be moderate quality.

### Sensitivity analysis and assessment of publication bias

Based on the prior definition, there were only three studies with a low risk of bias [12, 13, 16], so we did not conduct the sensitivity analysis based on the risk of bias. Sensitivity analyses of primary outcomes using the fixed effect model yielded stable overall results. Consider that each outcome included fewer than 10 studies, there were insufficient data for any publication bias analysis.

## Discussion

This meta-analysis aimed to assess the efficacy and safety of magnesium sulfate as an adjuvant in epidural bupivacaine in providing postoperative analgesia. We found that epidural bupivacaine combined with magnesium sulfate could prolong the time for first rescue analgesics. Furthermore, the addition of magnesium sulfate could reduce the number of patients who need rescue analgesics and requirement for rescue analgesics without adverse events. In addition, our meta-analysis showed that the incidence of shivering was lower with bupivacaine-magnesium sulfate than bupivacaine alone. Nevertheless, duration of motor blockade was significantly prolonged in group magnesium sulfate.

We found that many studies did not adequately report randomization methods. Five studies did not describe the generation of random sequences in detail [12, 14, 15, 19, 22], two studies did not describe the methods of allocation concealment [17, 18], three studies did not describe whether the anaesthetists were blind for this study design [14, 15, 20] and four studies did not report in detail their blind assessment of the outcomes [14, 15, 20, 21]. Only one studies reported clinical trials registration [12] and we were not clear about the risk of selective outcome reporting bias. We judged evidence for the time to the first request for rescue analgesics, the number of patients required rescue analgesics, requirement for rescue analgesia and duration of motor block to be very low certainty, and evidence for adverse events to be moderate quality. The low quality of evidence for primary outcomes was largely due to significant heterogeneity among studies and imprecision of the result. The significant heterogeneity might be explained by the differences in the types of surgery performed, the doses and manners of magnesium sulfate administered, and the postoperative pain management models. However, results of subgroup analyses on manners of magnesium administered did not appear to explain heterogeneity and we found that high levels of statistical heterogeneity still remained in both subgroups in each analysis. The surgery types varied between studies to include: cesarean section, unilateral thoracic, spinal, abdominal, orthopaedic. These differences may contribute to inconsistency and reduce the overall applicability of the evidence.

Whether the administrative route is intravenous, epidural, or intrathecal, the actual site of action of magnesium sulfate is probably at the spinal cord NMDA receptors. The analgesic effect primarily depends on Mg2+ blocks inward current flow through ion channels linked to NMDA receptors [23]. Although a previous systematic review found that intravenous administration of magnesium sulfate in orthopedic surgery could reduce postoperative requirement for postoperative analgesics and adverse events such as vomiting, nausea, and shivering [24], the role of intravenous magnesium sulfate was controversial. Some studies had found that intravenous magnesium failed to improve postoperative pain in gastrointestinal surgery [25] and in a pediatric population undergoing tonsillectomy [26]. The reason may be the limited ability of magnesium ions to penetrate the blood-brain barrier [27], so it seems plausible that epidural or intrathecia magnesium might be more effective. An earlier meta-analysis focused on cesarean section revealed that the additional neuraxial magnesium sulfate exerted significant effects on prolonging the duration of neuraxial anesthesia, reducing postoperative pain scores and decreasing requirement for postoperative analgesics, and was therefore in broad similar to our findings [28]. Furthermore, our findings are similar with the findings in a recent published meta-analysis, where the authors reported a reduction of the need for postoperative rescue analgesics in pediatric when magnesium was added to local anesthetics for caudal anesthesia [29].

In addition, magnesium sulfate has been compared with other epidural adjunct analgesic drugs. One study that evaluated the effects of epidural magnesium sulfate versus dexmedetomidine, and found that the time from epidural medication to first rescue analgesics was longer in dexmedetomidine group, duration of sensory and motor blockade was significantly prolonged in group dexmedetomidine, but risk of sedation increased and there was fall in the mean pulse rate in group dexmedetomidine [15]. Radwan et al. compared magnesium sulfate with fentanyl [21] and Mohammad et al. compared magnesium sulfate with clonidine [14] found the effect of magnesium sulfate on postoperative pain to be comparable to that of the other drugs.

In this meta-analysis, the addition of magnesium sulfate significantly prolonged the duration of motor block. The mechanism might be that magnesium inhibits the motor endplate release of acetylcholine due to inhibition of calcium-dependent channels [30, 31]. As for adverse effects, our meta-analysis showed that the incidence of shivering was lower in magnesium sulfate group. The reason might be that perioperative magnesium supplementation prevented the postoperative hypomagnesaemia and decreased the incidence of postoperative shivering [32].

There are some limitations to this study. Firstly, our meta-analysis demonstrates efficacy of epidural bupivacaine combined with magnesium sulfate when compared with bupivacaine alone in providing postoperative analgesia. Even if It is possible that our findings cannot be interpreted as truly positive because of the small sample sizes and the low quality of evidence assessed by GRADE framework; Secondly, We found that many studies did not adequately report randomization methods; Third, we didn’t assess publication bias due to the limited number of studies; Fourth, our findings showed high heterogeneity among studies, especially the existing clinical heterogeneity, such as types of surgery performed, the doses and manners of magnesium sulfate administered, and the postoperative pain management models.

## Conclusion

In conclusion, This meta-analysis revealed that magnesium suifate as an adjuvant of epidural bupivacaine improves postoperative analgesia. However, we rated the quality of evidence to be very low because of high heterogeneity, imprecise of results and small sample sizes. Furthermore, further large high-quality trials are still needed to confirm the effects of magnesium sulfate on postoperative analgesia.

## Data Availability

All data generated or analysed during this study are included in this published article.

## Appendix

### Search strategy

#### Cochrane Central Register of Controlled Trials

#1 MeSH descriptor: [Analgesia, Epidural] explode all trees.

#2 MeSH descriptor: [Anesthesia, Epidural] explode all trees.

#3 (epidural* or peridural* or subarachnoid* or extradural* or neuraxial*).

#4 (#1 or #2 or #3).

#5 magnesium OR MgSO4 OR Mg OR MS OR magnesium sulfate or magnesium sulphate.

#6((randomized controlled trial or controlled clinical trial).pt. or randomized.ab. or placebo.ab. or clinical trials as topic.sh. or randomly.ab. or trial.ti.) not (animals not (humans and animals)).sh.

#7 #4 and #5 and #6.

#### MEDLINE (OvidSP)

1. (Epidural* or peridural* or extradural* or subarachnoid* or neuraxial* or (Anesthesia, Epidural or Analgesia, Epidural)).af.
2. magnesium OR MgSO4 OR Mg OR MS OR magnesium sulfate or magnesium sulphate
3. ((randomized controlled trial or controlled clinical trial).pt. or randomized.ab. or placebo.ab. or clinical trials as topic.sh. or randomly.ab. or trial.ti.) not (animals not (humans and animals)).sh.
4. 1 and 2 and 3

#### Embase (OvidSP)

1. (epidural or peridural or extradural or subarachnoid or neuraxial) and (an?esth or analg) or (epidural anesthesia or epidural analgesia)
2. magnesium OR MgSO4 OR Mg OR MS OR magnesium sulfate or magnesium sulphate
3. (placebo.sh. or controlled study.ab. or random*.ti,ab. or trial*.ti,ab. or ((singl* or doubl* or trebl* or tripl*) adj3 (blind* or mask*)).ti,ab.) not (animal not (human and animal)).sh.
4. 1 and 2 and 3

## Abbreviations

CI: Confidence intervals
NMDA: N-methyl-D-aspartate
PRISMA: Preferred reporting items for systematic reviews and meta-analyses
RCTs: Randomized controlled trials
RR: Relative risk
SD: Standard deviation
SMDs: Standardized mean differences
